# Dimensionally reduced machine learning model for predicting single component octanol–water partition coefficients

**DOI:** 10.1186/s13321-022-00660-1

**Published:** 2023-01-19

**Authors:** David H. Kenney, Randy C. Paffenroth, Michael T. Timko, Andrew R. Teixeira

**Affiliations:** 1 Department of Chemical Engineering, Worcester Polytechnic Institute, Worcester, MA 01609 USA; 2 Department of Mathematical Sciences, Worcester Polytechnic Institute, Worcester, MA 01609 USA

**Keywords:** Molecular formula, Feature engineering, Model optimization, *LogP*

## Abstract

MF-LOGP, a new method for determining a single component octanol–water partition coefficients ($LogP$) is presented which uses molecular formula as the only input. Octanol–water partition coefficients are useful in many applications, ranging from environmental fate and drug delivery. Currently, partition coefficients are either experimentally measured or predicted as a function of structural fragments, topological descriptors, or thermodynamic properties known or calculated from precise molecular structures. The MF-LOGP method presented here differs from classical methods as it does not require any structural information and uses molecular formula as the sole model input. MF-LOGP is therefore useful for situations in which the structure is unknown or where the use of a low dimensional, easily automatable, and computationally inexpensive calculations is required. MF-LOGP is a random forest algorithm that is trained and tested on 15,377 data points, using 10 features derived from the molecular formula to make $LogP$ predictions. Using an independent validation set of 2713 data points, MF-LOGP was found to have an average $RMSE$ = 0.77 ± 0.007, $MAE$ = 0.52 ± 0.003, and ${R}^{2}$ = 0.83 ± 0.003. This performance fell within the spectrum of performances reported in the published literature for conventional higher dimensional models ($RMSE$ = 0.42–1.54, $MAE$ = 0.09–1.07, and ${R}^{2}$ = 0.32–0.95). Compared with existing models, MF-LOGP requires a maximum of ten features and no structural information, thereby providing a practical and yet predictive tool. The development of MF-LOGP provides the groundwork for development of more physical prediction models leveraging big data analytical methods or complex multicomponent mixtures.

**Graphical Abstract:**

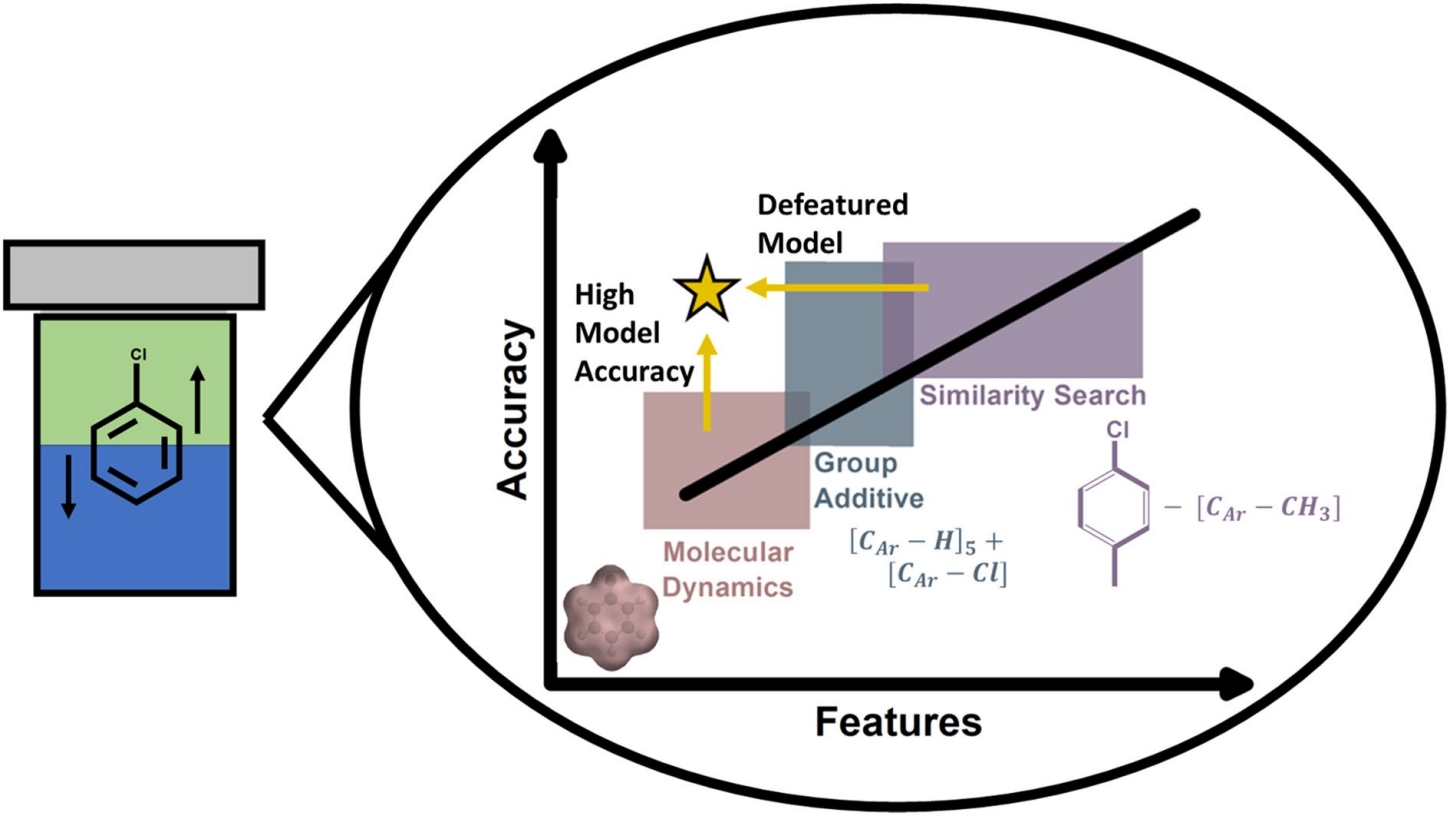

**Supplementary Information:**

The online version contains supplementary material available at 10.1186/s13321-022-00660-1.

## Introduction

The n-octanol/water partition coefficient (${P}_{OW}$) of a compound is a physical property that quantifies its lipophilicity relative to its hydrophilicity [1]. Partition coefficients play a determining role in the environmental fate and transport of pollutants [2–4]. For example, the soil sorption of heavily halogenated compounds, or “forever chemicals,” is strongly influenced by their n-octanol/water partition coefficients, with higher values associated with strong soil sorption [5]. In its base 10 log form($LogP$), partitioning behavior is an important factor in drug development and pharmacokinetics, where low values of $LogP$ are associated with greater bioavailability [6]. For example, Lipinski et al. included partitioning behavior in the “Rule of Five” stating that drugs must have $LogP$ values less than 5 to be orally active [7].

Thermodynamically, the partitioning of a solute between two phases is defined by chemical potential (${\mu }_{n,i}$) [8]:

1 $$\begin{array}{c}{\mu }_{n,i}={\mu }_{n}^{o}+RTln\left({a}_{n,i}\right) \end{array}$$

where ${\mu }_{n}^{o}$ is the chemical potential of compound $n$ at a given reference state (often taken to be pure liquid $i$), ${a}_{n,i}$ is the activity of $n$ in phase $i$, $R$ is the ideal gas constant, and $T$ is the absolute system temperature. If the system is assumed to be dilute, the activity can be modeled as ${a}_{n,i}={\gamma }_{{X}_{n},i}{\phi }_{n,i}$ where ${\phi }_{n,i}$ is the volume fraction of compound $n$ in phase $i$, and ${\gamma }_{n,i}$ is the activity coefficient which approaches unity for an ideal solution. At chemical equilibrium, the chemical potential of compound $n$ must be equal in all phases (i.e., ${\mu }_{n,i=\alpha }= {\mu }_{n,i=\beta }$ for phases a and b, which can be the organic and aqueous phases in an octanol–water system), and the partition coefficient becomes [8, 9]:

2 $$\begin{array}{c}{\mathrm{log}}_{10}\left({P}_{ow,n}\right)={\mathrm{log}}_{10}\left(\frac{{\left[n\right]}^{oct}}{{\left[n\right]}^{w}}\right)=\frac{{\mu }_{n,w}}{ {\mu }_{n,oct}}\end{array}$$

where the $ow$ subscript on $P$ denotes partitioning between octanol and water phases.

As an experimentally measured thermodynamic property, partition coefficients can be measured via the shake flask and slow-stir methods [10, 11]. In both cases, octanol and water are placed in a vessel, into which a small quantity of the compound of interest is injected. Samples are extracted and analyzed after equilibrium is reached. While these methods are the standard across the field, uncertainty is introduced if the mixtures have not yet reached equilibrium when the samples are extracted or in cases where microemulsions have formed [11]. According to the Organization for Economic Co-Operation and Development (OECD), the shake-flask method has a minimum standard deviation of 0.3 log units [10]. However, in practice and for a range of conditions consisting of varying temperature, pH, etc., the standard deviation ranges from 0.01–0.84 log units, often with similar magnitude of the averaged value itself [12]. In addition, the shake-flask and slow-stir methods are labor intensive, require off-line mixture analysis, and are hence costly. In an effort to address the lack of accuracy, researchers have advocated the use of microfluidics and improved *in-situ* analyses such as in-line UV or NMR to increase the speed and accuracy of measurements while reducing the generation of waste [13–15]. Unfortunately, microfluidic methods and in situ analysis requires specialized equipment that is not available in most labs. Both experimental strategies also require isolation of the pure compound, which may not be readily available, particularly in exploratory and discovery studies. Low-cost and rapid methods are needed to estimate $LogP$ when experimental measurement is not possible.

Since the 1960s, mathematicians and computer scientists have developed numerous regression methodologies that are now collectively termed *machine learning*. [16] These techniques range in complexity from simple linear regressions to neural networks, all of which are models that attempt to find the ideal relationship between independent and dependent variables. Regression techniques have been adopted by many fields [17–19], and starting in the 1970’s, applied to the prediction of partition coefficients by Rekker and coworkers [20]. By using an expansive database curated by Hansch and Leo [20]. Rekker et al. fit a linear regression model to structural fragments representing specific and well-defined portions of molecules to correlate molecular structure to experimental partition coefficients [19]. This model sparked the rapid development of regression algorithms that took structural inputs and produce physical property outputs, culminating in the Calculated LOGP (CLOGP) model that was the first model to have sufficient fragments for accurate predictions of realistic molecules [21].

Since these early efforts, numerous models have been developed to either introduce a new method or expand upon previous capabilities and accuracy [6, 22–28]. These models can be generalized by their features into three categories. (1) Molecular Simulation models, such as iLOGP [23],MLOGP [23], and ALOGPS [24] use physical structure to approximate electron densities, molecular size, and other topology and energetic insights [29]. In an independent study, MLOGP and ALOGPS have reported root mean square errors ($RMSE$) of 2.03 and 1.02 (log units), respectively, when predictions are compared with experimental measurements [22]. The greater accuracy of ALOGPS compared with MLOGP is explained by differences in model complexity (i.e., linear regression vs neural network) and differences in the quantity and complexity of features. (2) Fragment Additive Models, such as CLOGP [21], XLOGP2 [25], and WLOGP [26] break a molecule into a family of structural fragments and then calculate the $LogP$ using contributions from each fragment [30]. Reported $RMSE$ for XLOGP2 and CLOGP are 1.80 and 1.23 on the same independent analysis as the molecular simulation models [22]. The XLOGP2 algorithm was designed to fit 100 different atom/fragment types; the original CLOGP algorithm only had 58 learned constants but has since been updated to cover nearly 400 different fragments [31]. (3) Similarity Search algorithms are like (1) and (2) but have a fundamental difference in how the algorithm is initiated. The previous algorithms are most often the weighted sum of contribution across atoms, fragments, and other calculated properties; these calculations are not initialized by the structure of the molecule in any way. For similarity search algorithms like XLOGP3 [6] and KOWWIN [27], the molecule in question is compared to known compounds within a database and the experimental $LogP$ value of the most similar compound is used as an initial estimate the for the $LogP$ value. This rough estimate is then refined by applying correction factors to transform the reference compound to the one in question. The independently reported $RMSE$ reported for XLOGP2 is 1.80, whereas XLOGP3 was 1.08 log units [22]. Introducing the similarity search improved the accuracy of the XLOGP algorithm by 40%, and the accuracy improvement scales with the degree of similarity between the reference substance and the one being modeled [6].

Each of these regression methods, either directly or indirectly, require chemical structure as the primary model input which is then parsed into model specific features within the algorithm. In many cases, the exact chemical structure is known, and its requirement as an input to the algorithm is not a major problem. In some cases, however, the exact chemical structure of a compound is unknown, rendering existing $LogP$ methods ineffective. One such example occurs when dealing with big analytical data sets that do not resolve molecular structure, such as those arising from analysis of a complex mixture using mass spectrometry methods such as Matrix Assisted Laser Desorption Ionization [32] or Fourier Transform Ion Cyclotron Resonance Mass Spectroscopy (FT-ICR-MS) [33]. In these cases, the analysis provides molecular formulas and some measure of the relative abundance of components comprising the mixture, but without structural information. Another example where molecular structure may not be available is drug discovery. $LogP$ plays an important role in development of new drugs, since this value determines what methods are available for delivery or if the molecule is sufficiently bioavailable to achieve therapeutic effect. Using computer models to predict drug efficacy of theoretical pharmaceutical candidates is becoming increasing popular; the use of an automated, defeatured machine learning algorithm that does not require complex structural information can decrease computational costs to scan the multidimensional discovery space to identify drugs that partition in ways with favorable pharmacokinetic properties [34, 35].

For the situation in which molecular formula is the only known information, the number of different types of atoms present in the molecule is a natural set of features. For hydrocarbons, only three linearly independent features are possible: the number of carbon and hydrogen atoms and the H/C ratio. Molecular weight and double bond equivalents are two linear combinations that can be added to the feature matrix. More complex organic compounds that bear heteroatoms (N, O, S, P, F, Cl, Br, and I are most common) permit a corresponding increase in the number of features, but at the cost of much greater chemical complexity compared with simple hydrocarbons. Compared to methods that use hundreds of features, designing a model that can produce similar results with at most tens of features is a computational challenge. As such, successfully reducing predictive models from hundreds to tens of features has the potential to stimulate interest in lean models that retain predictive accuracy, avoid over fitting, and are easier to implement and use than existing models.

In this paper, we evaluate the accuracy of six machine learning regressions including linear regression, random forest, and k-nearest neighbors to predict organic compound $LogP$ values from elemental formulas. The six models were all trained, validated, and tested using a mined data set consisting of 18,091 data points available in the open literature. The resulting *Molecular Formula*-$LogP$ model, or MF-LOGP, can be utilized for organic molecules and is especially useful when molecular formula is the only available model input.

## Methods

### Data collection and preparation

A combination of databases, literature repositories, and web scraping methods was used to generate an initial dataset consisting of 24,047 $LogP$ values as outlined in Table 1, [1, 36–38]. The data was filtered to remove inorganic compounds and duplicate values, resulting in a dataset consisting of 18,091 data points. *PubChemPy* [39] and *CIRpy* [40] Python packages were used to add missing molecular formulas and SMILES strings.

**Table 1:** References used to compiless the initial dataset

Source	Number of data points	References
Sangster	628	[1]
Mansouri et al	10,273	[36]
PubChem	9571	[37]
National Cancer Institute	3575	[38]

The final curated dataset often contained multiple $LogP$ values for a single molecular formula. These were due to either, (1) multiple experimental values reported for a unique chemical compound (n = 14,143 unique compounds of 18,091 data points), or (2) isomers that presented with the same molecular formula but unique compounds and $LogP$ values (n = 7098 unique molecular formula of 14,143 unique molecular compounds). The first introduced experimental variance so that the model is more robustly trained. The second accounts for natural deviations in $LogP$ present among isomeric species with 2166 of the 7098 unique molecular formula contain at least two isomers. Since structural information is not needed on the front end of the MF-LOGP algorithm, the dataset must include multiple isomers to be robust enough for the algorithms to draw conclusions to accurate $LogP$ predictions and can further only be as accurate as these the natural deviation of $LogP$ values. As shown in Additional file 1: Figure S18, isomeric species generally vary between 0 and 2.78 log units, with an average deviation of 0.46 log units.

### Feature engineering

The predictive method considered here uses molecular formula as the sole input, then parses three types of features. The first type is the number of each atom present in the molecule (C, H, N, O, S, P, F, Cl, Br, I), which were determined from molecular formulas using chemparse in Python [41]. Additional features can also be expressed as the fractional content of each atom relative to the carbon content in the molecule, these are linearly independent additions to the feature matrix. The next two features are linear combinations of the first ten features (i.e. number of atoms), with practical implications as descriptors of molecular structure. The second feature type is the molar weight ($$M{W}_{n}$$) of compound $$n$$, which is determined by summing the products of the number of atoms of each element ($${X}_{n,m})$$ and their atomic weight ($${w}_{m}\left[=\right]g/mol)$$:

3 $${MW_{n} = \sum\limits_{{m = 1}}^{{M - 11}} {X_{{n,m}} }\cdot
w_{m} }$$

⋅where $X\in {M}_{N,M}({\mathbb{R}})$ is the two-dimensional feature matrix where the vector space is defined by compound indices (1 $\le n\le N$) and feature indeces (1 $\le m\le M$). The first ten columns (1 $\le m\le$ 10) represent the number of atoms per elements (C, H, N, O, S, P, F, Cl, Br, I). The 11 additional features are transformations of the first ten, representing the elemental ratios, MW (Eq. 3) and DBE (Eq. 4). Third, the double bond equivalence ($DBE$) can be calculated as:

4 $$\begin{array}{c}DB{E}_{n}={X}_{n,m=C}-\frac{{X}_{n,m=H}+{X}_{n,m=Halogens}}{2}+\frac{{X}_{n,m=N}}{2}+1 \end{array}$$

Here, ${X}_{(n,m=C)}$, ${X}_{(n,m=H)}$, ${X}_{(n,m=N)}$ are the number of atoms of carbon, hydrogen, and nitrogen within the molecular formula for compound ${X}_{n}$. ${X}_{(n,m=Halogens)}$ is the sum of all halogen atoms within compound ${X}_{n}$. *DBE* is the sum of the number of rings, double bonds, and triple bonds (multiplied by two) that appear a structure. For example, the $DBE$ of benzene (${C}_{6}{H}_{6}$) is 4, while that of cyclohexyne (${C}_{6}{H}_{8}$) is 3.

#### Functional groups

The goal of MF-LOGP was to predict partition coefficients without knowledge of molecular structure, including the presence of functional groups. While functional groups can undoubtedly increase accuracy by accounting for chemical behavior that results from specific orientation of the atoms, MF-LOGP explicitly omits these features. Future model improvements may account for functional group features by either allowing for the addition of functional groups or more generalized through chemometrics-derived molecular signatures such as infrared spectra. However, to test the limits of MF-LOGP, predictions were compared to their experimental partitioning, then grouped by functional groups present within each molecule during post-analysis. The occurrence of a given functional group was determined from the analysis of the corresponding SMILES string using the *RDkit* [42] package available in Python and then one-hot-encoded (1 = present, 0 = not present) the functional group presence into the dataset. The most common functional groups present in the dataset were aromatic, carbonyl, and alcohol groups. Additional file 1: Figure S1 provides a bar plot for the occurrences of each functional groups within the dataset.

### Model selection

Six commonly used regression models were chosen and evaluated for accuracy in this study: multivariate linear regression (MLR), ridge regression (RR), lasso regression (LR), random forest regression (RFR), gradient boosting regression (GBR), and k-nearest neighbor regression (KNNR).

#### Multivariate Linear Regression (MLR)

Linear regression is the simplest available form of correlation, and it has been used frequently for predictions of $LogP$. [6, 21, 23, 25–27] For this reason, MLR serves as the baseline performance metric for all other models. MLR is used to calculate the predicted $LogP$ of given compounds (${\widehat{y}}_{n}$) and can be described as:

5 $$\begin{array}{*{20}c} {\hat{y}_{n} = \sum\limits_{{m = 1}}^{M} {X_{{n,m}} }\cdot\beta _{m} + \beta _{0} } \\ \end{array}$$

where ${\widehat{y}}_{n}$ is the predicted value for compound ${X}_{n}$, $\beta \in {M}_{1,M}({\mathbb{R}})$ is a matrix of trained best-fit coefficients for each model feature ($M$ = 21), and ${\beta }_{0}$ is the best-fit ordinal intercept. If additional feature engineering is not desired (i.e., elemental ratios, $MW$, $DBE$), the model is trained with ${X}_{m}=0$ for the ${m}^{th}$ unwanted feature such that ${\beta }_{m}=0$. To reach an optimal model, linear regression aims to minimize the residual sum of squares (RSS) over all data points in the training set ($N$) [43]:

6 $${RSS = \sum\limits_{{n = 1}}^{N} {\left( {y_{n} - \hat{y}_{n} } \right)^{2} } }$$

where ${y}_{n}$ and ${\widehat{y}}_{n}$ are the experimental and predicted $LogP$ values for each compound in the training set.

#### Ridge Regression (RR)

Ridge regression (RR) is like the MLR algorithm such that it fits Eq. 5; however, the values of the fitting coefficients (${\beta }_{j}$) are included as a penalty in the minimization function [44]. The modified sum of squares is presented as:

7 $$\begin{array}{*{20}c} {\begin{array}{*{20}c} {RSS_{{RR}} = \sum\limits_{{n = 1}}^{N} {\left( {y_{n} - \hat{y}_{n} } \right)^{2} } + \lambda \sum\limits_{{m = 1}}^{M} {\beta _{m}^{2} } } \\ \end{array} } \\ \end{array}$$

where $\lambda$ is an optimized weighting factor.

#### Lasso Regression (LR)

Lasso regression (LR), like RR, fits Eq. 5 and constrains the fitting coefficients; however, at larger values of $\lambda$, the constraint term can set coefficients to 0. In this sense, Lasso can act as feature selection method [45]. The definition of the LR minimization function is:

8 $$\begin{array}{*{20}c} {RSS_{{LR}} = \sum\limits_{{n = 1}}^{N} {\left( {y_{n} - \hat{y}_{n} } \right)^{2} } + \lambda \sum\limits_{{m = 1}}^{M} | \beta _{m} |} \\ \end{array}$$

#### Random Forest Regression (RFR)

The random forest regression (RFR) is based on the decision tree regression, a method that uses features as part of an if–then-else structure either for classification or regression purposes. The RFR is an ensemble of decision tree regressors, meaning that it consists of many individual decision trees and the predicted value is an averaged value across all trees. The RF model is represented as [46]:

9 $$\begin{array}{*{20}c} {\hat{y}_{n} = \frac{1}{B}\sum\limits_{{b = 1}}^{B} {T_{b} } \left( {X_{n} } \right)} \\ \end{array}$$

where ${\widehat{y}}_{n}$ is the ensemble prediction for input (${X}_{n}$). The final output is an average of individual decision trees (${T}_{b}$) over the total number of trees ($B$). The error is minimized by the splitting of nodes within each decision tree, and the model convergence when the squared error of predictions depicted by Eq. 6 is minimized.

#### Gradient Boosted Regression (GBR)

The gradient boosting regression (GBR) algorithm is like RFR, except that it builds decision trees one at a time, whereas RFR builds trees simultaneously and independent of other trees in the forest. GBR builds decision trees one at a time so that the subsequent tree can minimize the errors of the previous tree rather than minimize the errors of the dataset. The generalized form of the GBR algorithm is [46]:

10 $$\begin{array}{*{20}c} {\hat{y}_{n} = \sum\limits_{{b = 1}}^{B} {h_{b} } \left( {X_{n} } \right)} \\ \end{array}$$

In Eq. 10, the ${h}_{b}$ term is called a “weak” learner. A weak learner is often restricted in depth and provides very little insight as a solo learner. However, when each learner is then summed, an accurate predicted value (${\widehat{y}}_{n}$)is produced. The algorithm will converge when the RSS described in Eq. 6 is minimized between the known values and the values predicted by Eq. 10.

#### k-Nearest Neighbors Regression (KNNR)

The k-nearest neighbors’ regression (KNNR) algorithm measures the Euclidian distance between the point of interest and all points within the training set:

11 $$\begin{array}{*{20}c} {ED_{{j,n}} = \sqrt {\sum\limits_{{m = 1}}^{M} {\left( {X_{{j,m}}^{{train}} - X_{{n,m}} } \right)^{2} } } } \\ \end{array}$$

The Euclidian Distance between training molecule, $j$, and molecule of interest, $n$, is denoted as $E{D}_{j,n}$. It is a function of difference between each entry of the training feature matrix, ${X}^{train}$ and the feature matrix describing the compound(s) of interest ($X$). KNNR makes no assumptions about the distribution of data in $N$ dimensional space. Once all distances are calculated, the algorithm will average the $K$ nearest points to the point of interest [45].

12 $${\hat{y}_{n} = \frac{1}{K}\left( {\sum\limits_{{k = 1}}^{K} m in\left( {ED_{n} ,k} \right)} \right)}$$

where $K$ is the user-defined number of neighbors used to determine a predicted value. The $min\left(E{D}_{n},k\right)$ is the ${k}^{th}$ smallest Euclidian Distance between the compound of interest and the training points. The model converges once the Eq. 6 is minimized between the known and predicted values.

### Hyperparameter tuning

Depending on the data set, regression-based models are prone to identifying a local minima, thereby missing the true global minimum required for accurate predictions. Tuning the hyperparameters of a model helps ensure that it reaches the global minimum and provides the most accurate results. The hyperparameters for the six models were tuned by using *GridSearchCV* [45] function in Python. The optimization was performed with an eightfold cross validation. A summary of the base hyperparameters and the tuned hyperparameters can be found Additional file 1: Table S1.

### Model performance parameters

Three metrics were chosen to compare the six different models: root mean square error ($RMSE$), mean absolute error ($MAE$), and coefficient of determination (${R}^{2}$). These metrics are defined as:

13 $$\begin{array}{*{20}c} {\begin{array}{*{20}c} {RMSE = \sqrt {\frac{{\sum _{{n = 1}}^{N} (\hat{y}_{n} - y_{n} )^{2} }}{N}} } \\ \end{array} } \\ \end{array}$$

14 $$\begin{array}{*{20}c} {MAE = \frac{{\sum _{{i = 1}}^{N} |\hat{y}_{n} - y_{n} |}}{N}} \\ \end{array}$$

15 $$\begin{array}{*{20}c} {R^{2} = 1 - \frac{{\sum _{{i = 1}}^{N} (\hat{y}_{n} - y_{n} )^{2} }}{{\sum _{{i = 1}}^{N} (y_{n} - \bar{y})^{2} }}} \\ \end{array}$$

In these equations $N$ is the total number of samples, ${\widehat{y}}_{n}$ are predicted values, $\overline{y }$ is the average known value, and ${\mathrm{y}}_{\mathrm{n}}$ are the known values. Both $RMSE$ and $MAE$ quantify the accuracy of a given prediction; they differ such that $RMSE$ imposes a greater penalty for large outlier predictions. The $RMSE$ and $MAE$ values for a well-fit model should be close together and near zero, thus both will be reported in this analysis.

### Data split and training

For model development, 85% of the data (15,377 out of 18,091 data points) were used for training and 15% (2,714 out of 18,142 data points) was reserved for final test. The testing data was not used during any of the training and validating of the six models so that it could serve as a fair metric of the predictive power of the regressed models.

A 2 × 3 factorial experimental design was used to evaluate the ideal training procedure for each algorithm. This method trained each model with eight different combinations of additional features, cross validation, and tuned hyperparameters. Each combination of parameters was iterated 100 times, allowing the training data to be randomized while keeping the testing data independent. The experiment matrix can be seen in Additional file 1: Tables S2 and S3, and the performance parameters of each model combination are represented in Additional file 1: Figures S2–S4.

## Results and discussion

### Dataset discovery

**Fig. 1:**
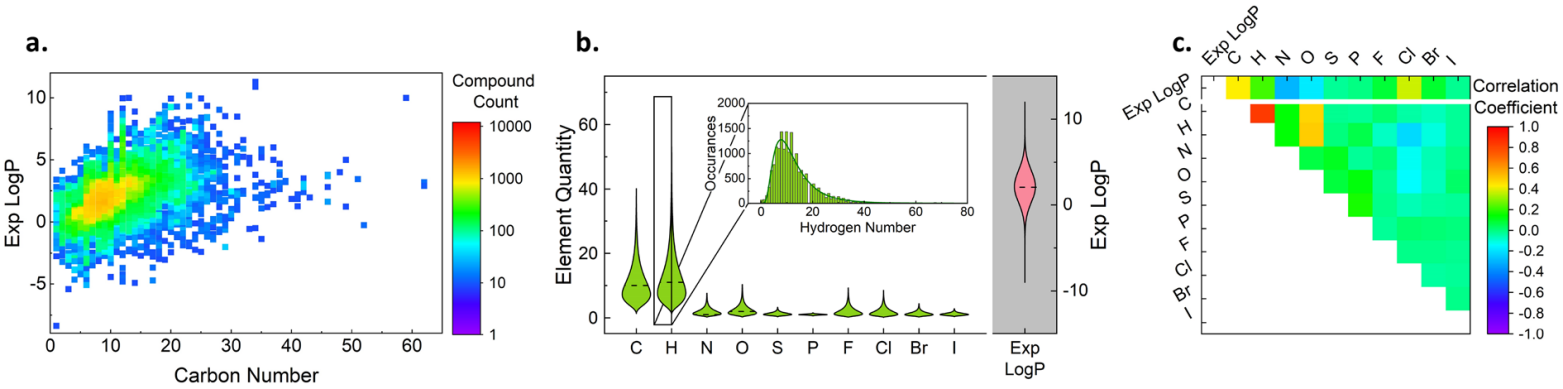
**a** Heat map of experimental partition coefficients as a function of carbon number. **b** Violin distribution plots of each elemental presence within the dataset. **c** Correlation matrix for feature-feature and feature-response correlations. The data set is highly populated from carbon numbers of 1–25 and $LogP$ values of  −2.5–5, this is region is expected to have the best performance. In addition to the quantity of data, compounds with chlorine, nitrogen and oxygen substitutions should lend to improved predictions as there are strong correlations to their partition coefficient

The first step of this study was to explore the dataset to understand its content and potential limitations for analysis. Figure 1 summarizes key features. Figure 1a contains a plot of $LogP$ values as a function of carbon number, showing that that the data is centered in the region defined by carbon numbers between 1–40 and log-partition coefficients of −5 to 7. The maximum carbon number present in the data set is 62, which defines the upper prediction limit for these models.

Figure 1b presents violin plots that are vertical and symmetrical representation of each feature’s histogram. The stand-alone histograms can be found in Additional file 1: Figures S5–S15. The molecules present in the data set are comprised primarily of carbon and hydrogen—with statistically greater hydrogen content than carbon, on an atomic basis—and typically contain zero of all other heteroatoms but can range up to 28 heteroatoms on a given molecule.

Figure 1c provides values of correlation coefficients among the atom number features themselves and between each element and values of $LogP$. The top row provides correlation coefficients between each of the atom number features and $LogP$. Among all features, the carbon and chlorine numbers have the greatest positive correlation coefficients (0.43 and 0.375) with $LogP$. Nitrogen and oxygen both have negative correlations (−0.30 and −0.19), consistent with the appearance of these atoms in polar functional groups such as alcohols and amines introduce hydrophilicity [47]. The correlation constants of all the remaining atoms (hydrogen, sulfur, phosphorous, and remaining halogens) are between 0 and 0.2, indicating weak correlation with $LogP$. Values of correlation coefficients shown in Fig. 1c are consistent the results of an F-test statistical analysis of feature importance, as shown in Additional file 1: Figure S16.

The remaining values of correlation constants capture feature-feature interactions. As expected, hydrogen is strongly correlated with carbon number. Interestingly, the oxygen number correlates more strongly with the carbon number than does nitrogen (0.48 vs 0.14), indicating that in this data set there is no correlation between the size of a compound and the number of nitrogen atoms, whereas oxygen tends to be present in larger ones. Parity plots of each correlation in Fig. 1c were plotted in Additional file 1: Figure S17 to further visualize the data.

Notably, of the 14,143 unique compounds in the curated dataset of $LogP$ values, experimental deviations ranging from 0–1.58 log units. Of the 7098 unique molecular formula, average standard deviations ranged from 0–2.87 with an average value of 0.46. The distribution of isomer standard deviations are displayed in Additional file 1: Figure S18.

### Model performances

**Fig. 2:**
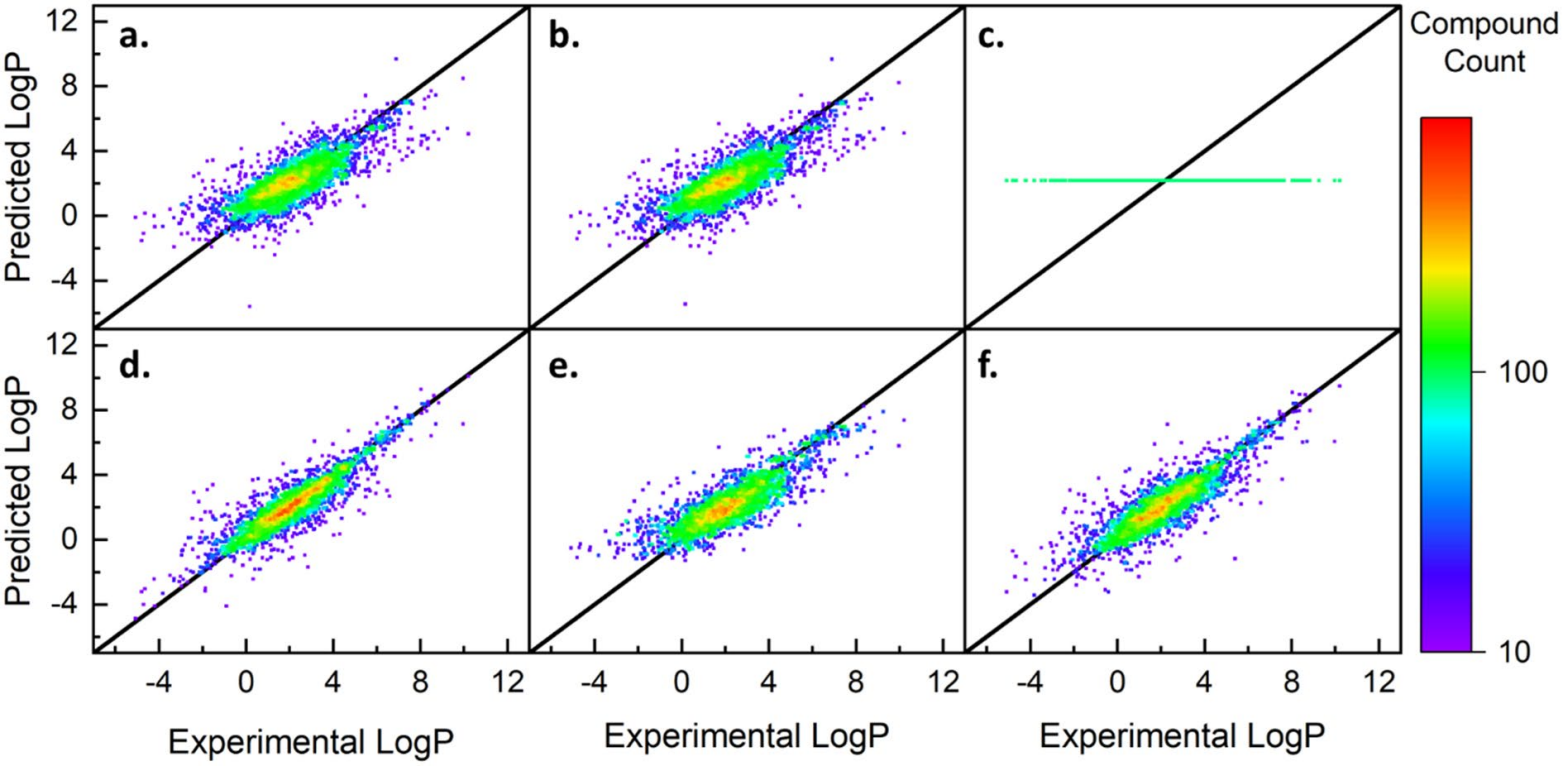
Parity density plots for experimentally determined and predicted values of the validation data (N = 3,076) for each of the six base regression models. The linear models, except for Lasso, appear to have a similar performance while the Random Forest shows a better visual fit and higher density of points along parity line. **a** Linear, **b** Ridge, **c** Lasso, **d** Random Forest, **e** Gradient Boosted, **f** k-Nearest Neighbors

The remaining 85% (15,377 of 18,091) of the data points not set aside for final testing were used to train and validate the six models according to the experimental design outlined. Specifically, 80% (12,301 of 15,377) of this data was used train the models and the remaining 20% (3076 of 15,377) was used to validate the performance. Parity density plots of the validation performances for all six default models are shown in Fig. 2. Visually, the tightness of fit around the black parity line and the yellow/red section in the middle indicate a good grouping of predictions. For a perfect model, the predictions would follow the solid black parity line with the highest density (red) from -1 to 6 and a decrease in density on both sides as consistent with the known distribution in Fig. 1a. To quantify the performance depicted in Fig. 2, the models were trained and validated 100 times, each time randomizing the remaining 80% of data used for training. Doing this identifies anomalies in the data or model development. The averaged findings of this analysis can be found in Table 2 and the standard deviations of each value in Additional file 1: Table S4. This evaluation was carried out for the eight experimental combinations outlined in Additional file 1: Tables S2 and S3. According to Additional file 1: Figures S2–S4, the default RFR (no cross validation, additional features, or hyperparameters) performed the best out of the eight combinations.

**Table 2:** Model performance parameters for all six default models for both training and validation data averaged over 100 experiments

	$\mathrm{RMSE}$		$\mathrm{MAE}$		${\mathrm{R}}^{2}$	
	Training	Validation	Training	Validation	Training	Validation
Linear	1.149	1.151	0.845	0.846	0.629	0.628
Ridge	1.149	1.151	0.846	0.847	0.629	0.628
Lasso	1.887	1.887	1.436	1.435	0.000	0.000
Random Forest	0.497	0.797	0.322	0.518	0.931	0.822
Gradient Boosted	0.961	0.988	0.702	0.718	0.741	0.726
Nearest Neighbors	0.743	0.905	0.502	0.616	0.845	0.770

Of the six models, three were linear (MLR, RR, LR) and three were non-linear (RFR, GBR, KNNR). For MLR and RR, identical performances were observed with $RMSE$ values of 1.151. The lasso regression was over-constrained and set all learned coefficients to zero. The RFR is shown to have the optimal performance compared to the other methods with a validation error of 0.797. According to the results in Table 1, the RFR and KNNR are both overfit as their training errors are much smaller than the validation error. Typically, this is a cause for concern because it tends to decrease the predictive nature of the model, but in this case, we see that RFR still outperforms all other models with an $RMSE$ 0.1 log units smaller than the closest model (KNNR).

Introducing hyperparameters in the training method either by themselves or in combination with cross validation or feature engineering produced a LR method that was comparable to MLR and RR (RMSE = 1.15 $\pm$ 0.008, MAE = 0.85 $\pm$ 0.007, R^2^ = 0.63 $\pm$ 0.005). For RFR, there was a slight improvement with feature engineering (RMSE = 0.78 $\pm$ 0.02, MAE = 0.50 $\pm$ 0.01, R^2^ = 0.83 $\pm$ 0.01), however these results are within error of the base model reported in Table 2. Therefore, the 10-feature RFR base model was chosen as the final MF-LOGP model for all further analyses. Additional file 1: Figure S19 and Table S4 show the validation parity density plots and error values of each of the six models with the addition of feature engineering.

### Comparing predictions by compound class

**Fig. 3:**
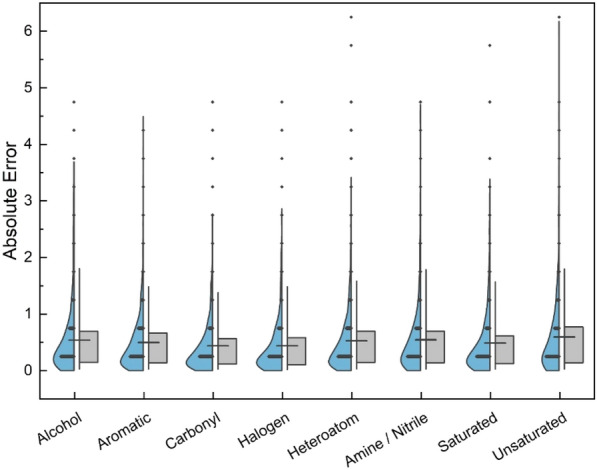
Violin (BLUE) and box (GREY) plots highlight the error distribution using MF-LOGP between different functional groups, demonstrating consistent model performance across all studied functional groups

Using the functional groups identification via SMILES strings, a more in-depth error analysis of MF-LOGP was performed. After training the MF-LOGP model, the absolute errors of each compound were calculated between their predicted and experimental values, then grouped based on functional group presence within a molecule. When multiple functional groups are present in the same molecule, the molecule was counted in multiple groupings. Figure 3 plots the errors in each group and represents them as vertical distributions coupled with box and whisker plots that highlight the percentile breakdown between the 5th (lower) and 95th (upper) percentiles.

**Fig. 4:**
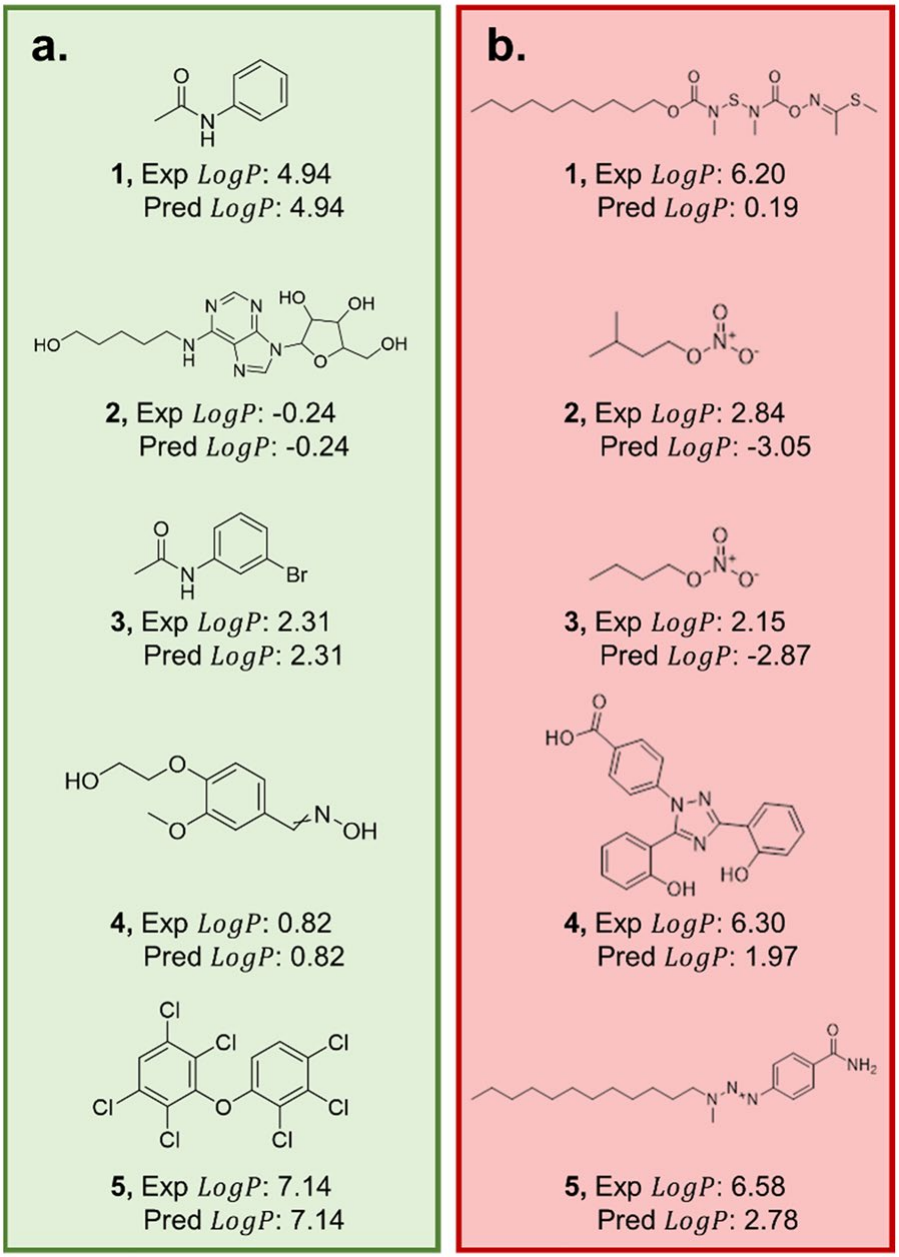
Structural representation of **(a)** the five compounds with the best predictions and **(b)** the five compounds with the largest difference between prediction and experimental value. The presence of halogens and more simply substituted aromatics lend to a more accurate prediction. More complex aromatic systems and ionized compounds offer less accurate predictions

According to the results represented in Fig. 3, most groups have similar distributions, with their averages falling near 0.52 log units, which was reported for the average absolute error for the overall model. Unsaturated compounds appear to have the broadest distribution, ranging from 0 to 6.5 log units, indicating the model fails to strongly predict some of this complexity introduced by double bonds, triple bonds, rings, and their isomers. According to Additional file 1: Figure S1, the alkene/alkyne groups are the least represented in the data, likely also contributing to skewed predictions. Individually, the largest averaged errors fall with unsaturated, amine/nitrile, and heteroatom groups (0.59, 0.54, and 0.53 log units). To put more context to these findings, 10 compounds with largest and smallest absolute errors were pulled from the dataset and had their structures identified. Figure 4 shows the structure of the five compounds from the validation set that yielded the largest and smallest individual absolute errors.

The complete set of structures are presented in Additional file 1: Figure S20, and their predictions along with expected values listed in Additional file 1: Table S6. Generally, the better predicted compounds appear to be halogenated compounds or aromatics with smaller substitution groups. The less accurate predictions appear to be long hydrocarbon compounds or aromatics with longer alkyl substitutions, as well as ionized compounds such as compound 2b or 3b in Fig. 4.

In addition to resolving error between compound classes, an additional determined source of error is extrapolating to compounds larger than that in the MF-LOGP dataset. The data used to train this model typically had a sum of non-hydrogen atoms less than 30. Additional file 1: Figure S21 shows the comparison of the MF-LOGP dataset and external data published by Ulrich et al [48] and Plante et al [49]. Notably, the sum of non-hydrogen elements is beyond the range of the MF-LOGP dataset. The errors on these datasets are shown in Additional file 1: Table S7 and as expected, were larger than the values reported for the MF-LOGP.

### Final test and external method comparison

**Fig. 5:**
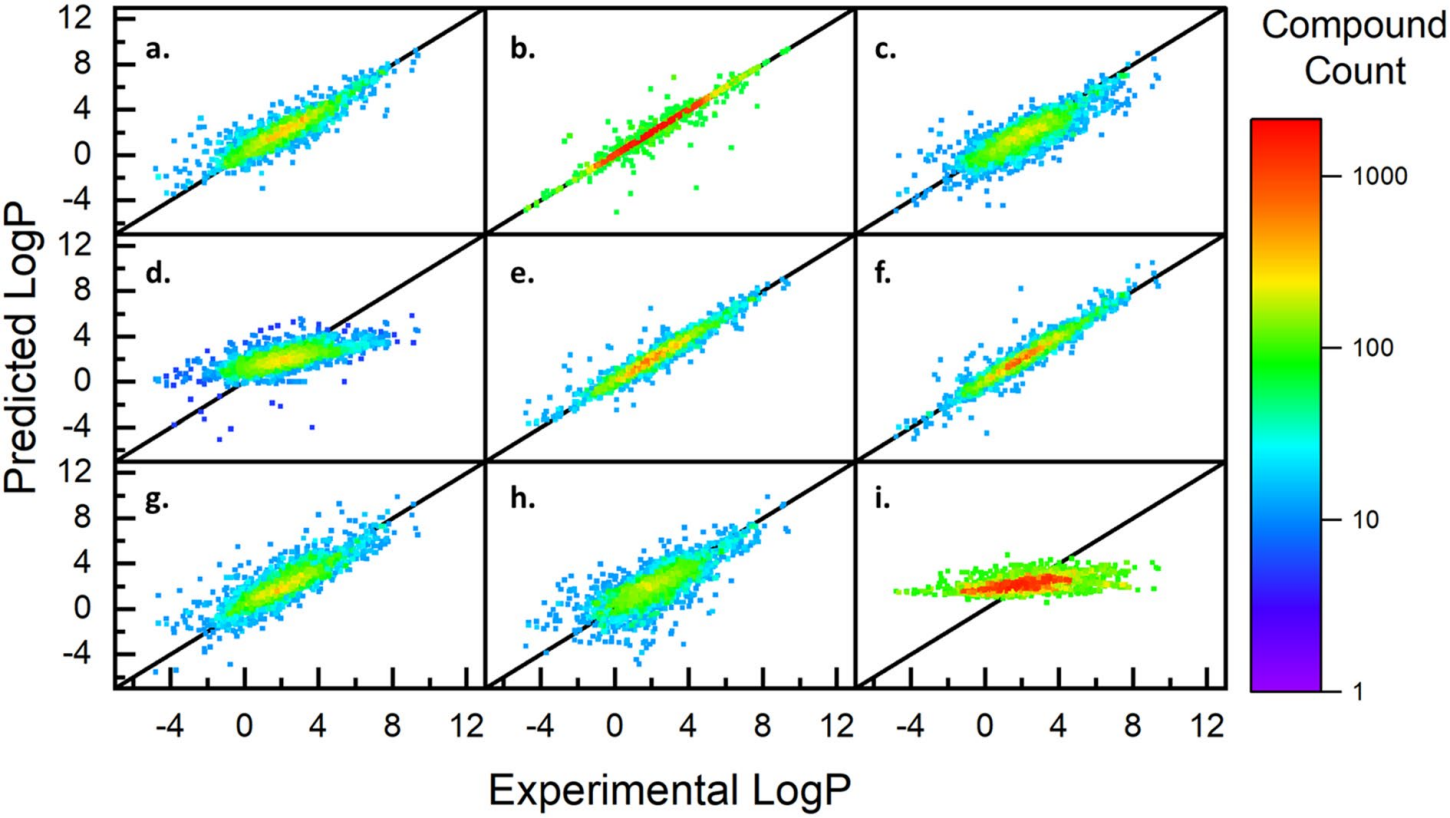
Parity density plots of final testing data (N = 2,713) for the deployed MF-LOGP algorithm as well seven peer-reviewed methods that have demonstrated strong predictive capabilities, but all require structural information. MF-LOGP outperforms four of the current models and has a close performance to the EPA’s KOWWIN algorithm. **a** MF-LOGP, **b** XLOGP3, **c** MLOGP, **d** iLOGP, **e** ALOGPS, **f** KOWWIN, **g** WLOGP, **h** SILICOS-IT, **i** AAM

The goal of this work is to develop a machine learned model that accurately predicts $LogP$ values using only features discerned from the molecular formula. Table 2 shows that the RFR achieves an $RMSE$ and $MAE$ of 0.797 and 0.518, respectively. Both parameters indicate superior performance to the base model (MLR), but do not yet provide insights to its capabilities with unknown compounds. For this, the 15% testing data (2714 out of 18,091 data points) were used to give an unbiased performance of MF-LOGP as well as eight other models currently published in literature as described in Table 3. Parity plots of predicted values relative to experimental values for each of these eight models are plotted in Fig. 5 and relevant performance parameters listed in Table 4.

**Table 3:** Names and descriptions of each model being used as a comparison

Model name	Model types	Number of features	References
MF-LOGP	Defeatured Atom Additive	10	N/A
XLOGP3	Similarity Search + Fragment	88	[6]
KOWWIN	Similarity Search + Fragment	400	[27]
WLOGP	Fragment	68	[26]
MLOGP	Molecular Simulation	13^*^	[23]
iLOGP	Molecular Simulation	2^*^	[23]
ALOGPS	Molecular Simulation	115^*^	[24]
SILICOS-IT	Fragment + Molecular Simulation	35	[28]
AAM	Simple Atom Additive	2	[22]

^*^Number of features within the molecular simulation models do not account for the original input of structural information

**Table 4:** Performance parameters for independent testing data on all models

	MF-LOGP	XLOGP3	MLOGP	iLOGP	ALOGPS	KOWWIN	WLOGP	SILICOS-IT	AAM
$RMSE$	0.77	0.42	1.08	1.54	0.47	0.67	0.94	1.20	1.64
$MAE$	0.52	0.09	0.78	1.07	0.30	0.40	0.68	0.87	1.23
${R}^{2}$	0.83	0.95	0.72	0.32	0.94	0.93	0.75	0.62	0.51

According to the results in Table 4, the MF-LOGP algorithm has a an $RMSE$, $MAE$, and ${R}^{2}$ of 0.77, 0.52, and 0.83 respectively. The errors reported in Table 4 give confidence that (1) the MF-LOGP algorithm is not overfitting in the training methods as the testing error is similar to the validation error, (2) MF-LOGP predictions are robust enough to predict within isomeric deviations which were typically 0.46 $\pm$ 0.44 log units (Additional file 1: Figure S18), and (3) MF-LOGP has similar performance to existing models that require significant structural knowledge such as KOWWIN and XLOGP3.

**Fig. 6:**
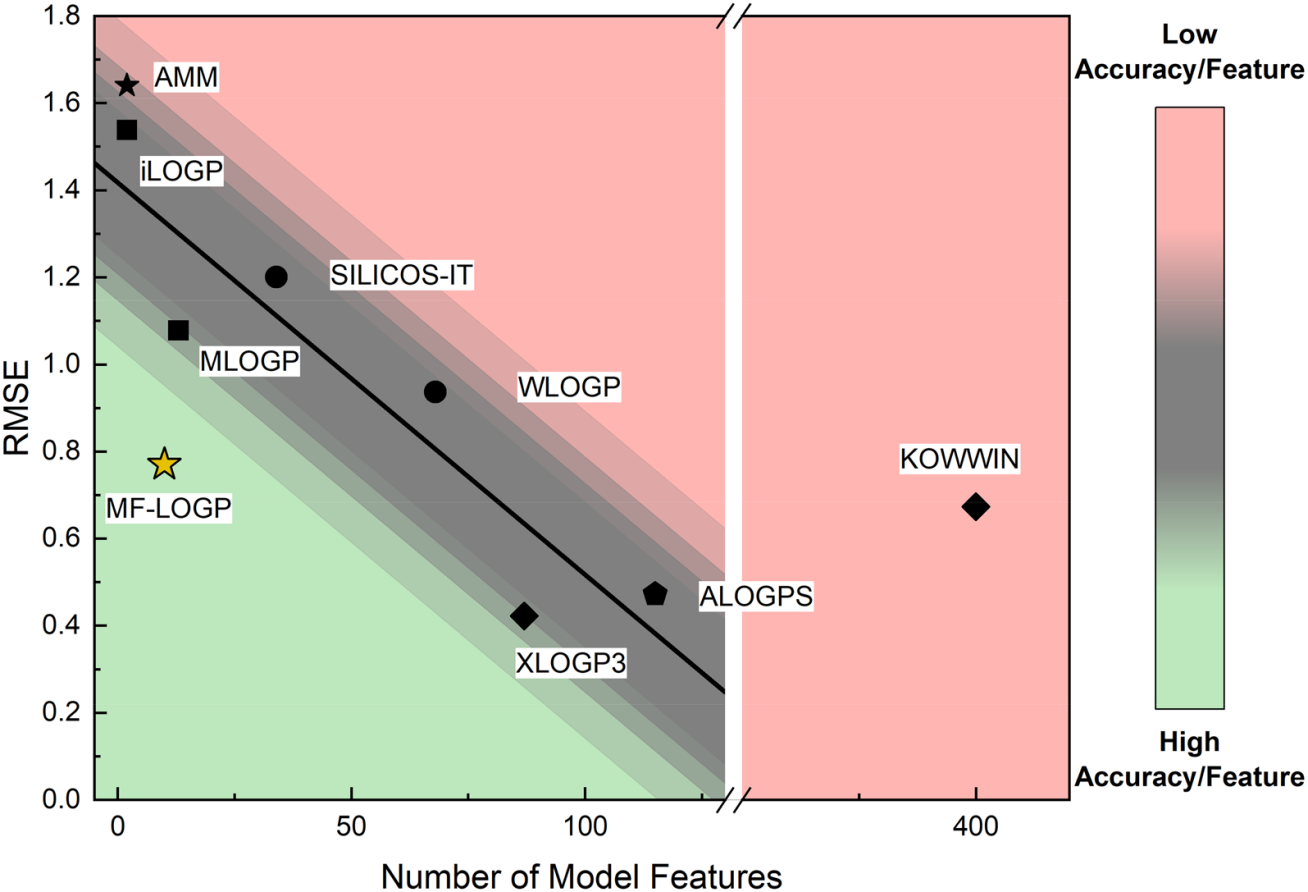
Scatter plot representing performance of MF-LOGP and published models on the final test data set (N = 2,713) as a function of the number of features required by the model. (■) Molecular simulations, (●) fragment/topological analysis, (◆) fragment additive + similarity search, (⬟) molecular simulation with neural network, (★) structurally independent atom additive. The bottom left corner represents the region represents high accuracy with the fewest number of features. The top right region represents models with high errors despite having substantial number of features

The results of from Fig. 5 and Table 4 are compiled into a final comparative analysis which is displayed in Fig. 6. The error associated with the independent test set was plotted as a function of the features used within each model. Within the plot, each model is categorized by the first model type listed in Table 3 for reach model and represented as symbols on the plot. Molecular simulation models are represented as squares (■), fragment additive models are shown as circles (●), similarity search algorithms are shown as diamonds (◆), SILICOS-IT – a combination of fragment and simulation additive—is denoted as a triangle (▲), and finally, the structurally independent atom additive models are denoted as stars (★). Three key observations are made clear immediately: (1) Literature models are improved by increasing the features that the model fits, (2) Existing model accuracy can be binned as similarity search > fragment additive > molecular simulation, and (3) MF-LOGP outperforms the structurally independent, simple regression proposed by Mannhold et al. [22] Two exceptions are observed. First, while moderately accurate (RMSE = 0.67), KOWWIN uses 400 features, much more than expected to achieve such accuracy. Second, ALOGPS outperforms WLOGP and is competitive with XLOGP3. ALOGPS leverages a neural network which lends users a more accurate prediction but has a hidden cost by requiring iterative molecular simulation simulations to calculate features based on molecular structure. Finally, MF-LOGP is shown in the region of high accuracy compared to models with a similar number of features. The KOWWIN algorithm utilizes 4000% more features compared to the MF-LOGP algorithm to account for most generalized fragment groups that appear in organic compounds. By eliminating structural information, MF-LOGP performs between 13–45% behind the similarity search and neural network algorithms. However, MF-LOGP performs 22–100% better than more traditional fragment and molecular simulation additive models. This confirms that while MF-LOGP is significantly reduced in both number of features and knowledge of features, it stands to be competitive against current partition coefficient models.

## Conclusion

Until this point, predictive methods have relied entirely on the structural identifiers of a compound to either define structural fragments that correlate with partitioning or are the starting point for thermodynamic simulations that use molecular simulations to calculate phase partitioning. Models have become increasingly accurate by using machine learning algorithms to more creatively define features based on molecular structure. They have particularly benefited from similarity matching, additive methods, and partition predictions. The work shown here, however, did so by relaxing the assumption that features must be derived from the molecular structure, and instead demonstrated that defeaturing a model to rely only on the molecular formula created a lean model that accurately predicted LOGP with errors comparable to model with an order of magnitude greater number of features that required rich structural information.

The MF-LOGP algorithm presented in this work breaks the curve for both feature and model complexity. A structurally informed model with only 10 independent features is expected to have an average $RMSE$ of 1.33, yet the structurally agnostic MF-LOGP model produces an averaged $RMSE$ of 0.77. This model is comparable to widely implemented methods such as KOWWIN model which contains 400 features. In addition to the impressive accuracy of this model, it does not rely on structural information, opening the door to future partitioning analyses of complex systems with unknown or unresolved molecular structures.

## Supplementary Information

**Additional File 1:** Supporting data for feature engineering, hyperparameter tuning, data splitting, and exploratory data analysis; model performances with feature engineering; and prediction capabilities of various compound classes.

## Data Availability

The datasets are available per citations referenced in Table 1. Two sets of code are made public through the Teixeira Research Lab GitHub Repository, https://github.com/TeixeiraResearchLab/MF-LOGP_Development-. The first code trains and reports error for a user-provided dataset using all models presented in this work. The second code is the final MF-LOGP algorithm, as trained and optimized above. All code was developed in Python using Anaconda.

## Abbreviations

DBE_n_: Double bond equivalents of compound n
ED: Euclidian distance
GBR: Gradient boosting regression
KNNR: K-Nearest Neighbor Regression
LogP: Natural Log of the N-Octanol/Water Partition Coefficient
LR: Lasso regression
MAE: Mean absolute error
MF-LOGP: Molecular Formula LogP Prediction Algorithm
min⁡(ED_n_,k): The kth Minimum Euclidian Distance
MLR: Multivariate linear regression
MW_n_: Molecular weight of compound n
P_OW_: N-Octanol/Water Partition Coefficient
RFR: Random forest regression
RMSE: Root mean square error
RR: Ridge regression
RSS: Residual sum of squares
SMILES: Simplified molecular input line entry system

## List of symbols

a_n,i_: Activity of Compound n In Phase i
B: Total Number of Trees
h_b_: “Weak” Learner
J: Total Number of Training Compounds
K: User-Defined Number of Neighbors
M: Total Number of Features
min⁡(ED_n,k_): The kth Minimum Euclidian Distance
n: Compound Indexer
N: Total Number of Compounds
R: Ideal Gas Constant (Units)
R^2^: Coefficient of Determination
T: Absolute Temperature (K)
T_b_: Final Output of Individual Trees
w_m_: Atomic Weight Of m^th^ Element in Molecular Formula
X_n_: Feature Matrix for Compound n
y_n_: Experimentally Determined Value of Partition Coefficient for Compound n
y_n_: Predicted Partition Coefficient for Compound n
y_n_: Averaged Known Partition Coefficients
β: Learned Feature Coefficients
γ_n,i_: Activity Coefficient for Compound n In Phase i
λ: Optimized Weighting Factor
ϕ_n,i_: Volume Fraction of Compound n in Phase i
μ_n,i_: Chemical Potential of Compound n in Phase i
μ^o^_n,i_: mical Potential of Compound n at A Given Reference State
