# Supplementary material for: Dimensionally reduced machine learning model for predicting single component octanol–water partition coefficients
Source: J Cheminform. 2023 Jan 19;15:9. doi: 10.1186/s13321-022-00660-1 (PMC9854055; doi:10.1186/s13321-022-00660-1)
Supplement: Supplementary file 1 — Additional File 1: Supporting data for feature engineering, hyperparameter tuning, data splitting, and exploratory data analysis; model performances with feature engineering; and prediction capabilities of various compound classes. [file 13321_2022_660_MOESM1_ESM.docx]

**Dimensionally Reduced Machine Learning Model for Predicting Single Component
Octanol-Water Partition Coefficients**

David Kenney ^a^, Randy C. Paffenroth^b^, Michael Timko^a^, Andrew R. Teixeira ^a,*^

1. Department of Chemical Engineering, Worcester Polytechnic Institute, Worcester, Massachusetts 01609, United States
2. Department of Mathematical Sciences, Worcester Polytechnic Institute, Worcester, Massachusetts 01609, United States

* Correspondence should be addressed to [arteixeira@wpi.edu](mailto:arteixeira@wpi.edu)

**Keywords:** Molecular Formula, Feature Engineering, Model Optimization

**Table of Contents**

[S2.2. Feature Engineering 3](#_Toc111018566)

[Figure S1. Functional group occurance within overall dataset 3](#_Toc111018567)

[S2.4. Hyperparameter Tuning 3](#_Toc111018568)

[Table S1. Model parameters for pre-defined and tuned models 3](#_Toc111018569)

[S2.6. Data Split and Training 3](#_Toc111018570)

[Table S2. 2x3 Factorial design options 3](#_Toc111018571)

[Table S3. 2x3 Factorial design matrix 4](#_Toc111018572)

[Figure S2. $\boldsymbol{RMSE}$ results for each model in the DoE matrix 4](#_Toc111018573)

[Figure S3. $\boldsymbol{MAE}$ results for each model in the DoE matrix 5](#_Toc111018573)

$\boldsymbol{HYPERLINK \backslash l "\_Toc111018575"}$Figure S4. $\mathbf{R}^{\mathbf{2}}$ results for each model in the DoE matrix 5

[S3.1. Dataset Discovery 6](#_Toc111018576)

[Figure S5. Carbon number occurances 6](#_Toc111018577)

[Figure S6. Hydrogen number occurances 6](#_Toc111018578)

[Figure S7. Nitrogen number occurances 7](#_Toc111018579)

[Figure S8. Oxygen number occurances 7](#_Toc111018580)

[Figure S9. Sulfur number occurances 8](#_Toc111018581)

[Figure S10. Phosphorous number occurances 8](#_Toc111018582)

[Figure S11. Fluorine number occurances 9](#_Toc111018583)

[Figure S12. Chlorine number occurances 9](#_Toc111018584)

[Figure S13. Bromine number occurances 10](#_Toc111018585)

[Figure S14. Iodine number occurances 10](#_Toc111018586)

[Figure S15. $\boldsymbol{LogP}$ number occurances 11](#_Toc111018587)

[Figure S16. F-test anaysis for feature importances 11](#_Toc111018588)

[Figure S17. Feature-feature and feature-response parity plot matrix 12](#_Toc111018589)

[S3.2. Model Performances 12](#_Toc111018590)

[Table S4. Averaged base perfomance parameters with standard deviation 12](#_Toc111018591)

[Figure 18. Histogram of molecular formula isomer partition coefficients 1](#_Toc111018592)3

[Figure 19. Parity plots for model performances with feature engineering 1](#_Toc111018592)3

[Table S5. Averaged feature-engineered perfomance parameters with standard deviation 1](#_Toc111018593)3

[S3.3. Comparing Predictions by Compound Class 14](#_Toc111018594)

[Figure S20. Top ten compounds with the largest and smallest absolute errors 1](#_Toc111018595)5

[Table S6. Comparison of predicted and experimental partition coefficients for the 10 worst predictions IUPAC Structure 1](#_Toc111018615)6

[Figure S21. Distribution of compounds against the quantity of non-hydrogen atoms 1](#_Toc111018615)7

[Table S7. Analysis of MF-LOGP using fully external datasets 1](#_Toc111018615)7

# **S2.2. Feature Engineering**

**Figure S1.** Bar plots of functional group occurrences within the dataset used for the training of the MF-LOGP algorithm. Each functional group presence was determined using each compounds unique SMILES string. It is readily apparent that nitrile, alkene, and alkyne functional groups are not readily present within the dataset.

# **S2.4. Hyperparameter Tuning**

**Table S1.** Pre-defined and tuned model parameters for each of the six models

| Model | Base Hyperparameters | Tuned Hyperparameters |
| --- | --- | --- |
| Linear | N/A | N/A |
| Ridge | ‘alpha’ = 1 | ‘alpha’ = 10 |
| Lasso | ‘alpha’ = 1 | ‘alpha’ = 0.0001 |
| Random Forest | ‘n_estimators’ = 100  ‘min_samples_split’ = 2  ‘min_samples_leaf’ = 1  ‘max_features’ = 10 | ‘n_estimators’ = 1000  ‘min_samples_split’ = 4  ‘min_samples_leaf’ = 2  ‘max_features’ = 6 |
| Gradient Boosted | ‘n_estimators’ = 100  ‘min_samples_split’ = 2 | ‘n_estimators’ = 500  ‘min_samples_split’ = 3 |
| k-Nearest Neighbor | ‘n_neighbors’ = 5  ‘leaf_size’ = 30 | ‘n_neighbors’ = 7  ‘leaf_size’ = 4 |

# **S2.6. Data Split and Training**

**Table S2.** Factorial design options for cross validation, feature engineering, and hyperparameter tuning

|  | **k-Fold Cross Validation** | **Feature Engineering** | **Hyperparameters** |
| --- | --- | --- | --- |
| $+$ | k = 8 | Additional features added | Tuned hyperparameters |
| $-$ | Randomized train/test split | No additional features | Base model parameters |

**Table S3.** 2x3 Factorial design of experiments. For each of the eight options the plus ($+$) and minus ($-$) symbols correlate to the options outlined above in Table S2

| **Experiment** | **k-Fold Cross Validation** | **Feature Engineering** | **Hyperparameters** |
| --- | --- | --- | --- |
| 1 | $-$ | $-$ | $-$ |
| 2 | $+$ | $-$ | $-$ |
| 3 | $-$ | $+$ | $-$ |
| 4 | $-$ | $-$ | $+$ |
| 5 | $+$ | $+$ | $-$ |
| 6 | $+$ | $-$ | $+$ |
| 7 | $-$ | $+$ | $+$ |
| 8 | $+$ | $+$ | $+$ |

**Figure S2.** Averaged root mean square error for each iteration of the 2x3 design of experiments matrix outlined in Table S3 for 100 iterations. The gradient boosting and lasso regressions are both largely impacted by the use feature engineering and hyperparameter tuning (experiments 4, 7, and 8). The random forest regression observes some fluctuation in performance but not outside of significance of the base performance ($RMSE$ = 0.797).

**Figure S3.** Averaged mean absolute error for each iteration of the 2x3 design of experiments matrix outlined in Table S3 for 100 iterations. The gradient boosting and lasso regressions are both largely impacted by the use feature engineering and hyperparameter tuning (experiments 4, 7, and 8). The random forest regression observes some fluctuation in performance but not outside of significance of the base performance ($MAE$ = 0.518).

.

**Figure S4.** Averaged mean absolute error for each iteration of the 2x3 design of experiments matrix outlined in Table S3 for 100 iterations. The gradient boosting and lasso regressions are both largely impacted by the use feature engineering and hyperparameter tuning (experiments 4, 7, and 8). The random forest regression observes some fluctuation in performance but not outside of significance of the base performance ($R^{2}$ = 0.822).

# **S3.1. Dataset Discovery**

**Figure S5.** Histogram of carbon number occurrences within the dataset

**Figure S6.** Histogram of hydrogen number occurrences within the dataset

**Figure S7.** Histogram of nitrogen number occurrences within the dataset

**Figure S8.** Histogram of oxygen number occurrences within the dataset

**Figure S9.** Histogram of sulfur number occurrences within the dataset

**Figure S10.** Histogram of phosphorus number occurrences within the dataset

**Figure S11.** Histogram of fluorine number occurrences within the dataset

**Figure S12**. Histogram of chlorine number occurrences within the dataset

**Figure S13**. Histogram of bromine number occurrences within the dataset

**Figure S14.** Histogram of iodine number occurrences within the dataset

**Figure S15.** Histogram of experimental $LogP$ number occurrences within the dataset

**Figure S16.** F-test statistical analysis indicating the importance of each feature in the prediction of partition coefficients


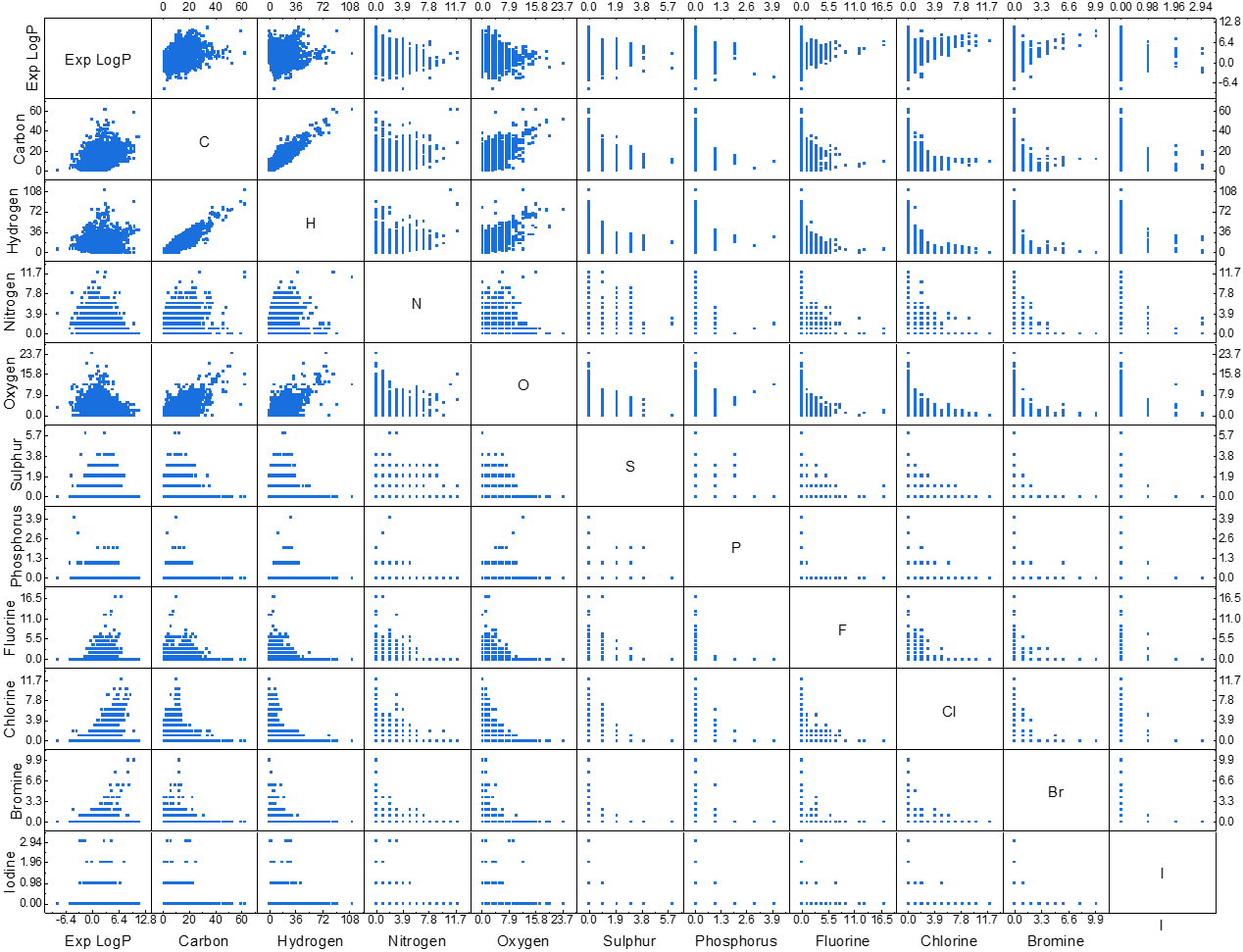


**Figure S17.** Scatter parity plots between model features and responses. These results model the correlation coefficients depicted in Figure 1. Between the features, there is a clear positive trend between carbon and hydrogen, carbon and nitrogen, and carbon and oxygen. For the features impact on the response predictions, there is a positive trend between with carbon, chlorine, and bromine. Nitrogen and oxygen have a slightly negative relationship with the response. The remaining features have seemingly little predictive capabilities.

# **S3.2. Model Performances**

**Table S4**. Averaged values for $RMSE$, $MAE$, and $R^{2}$ for each base model over 100 iterations of different training and validation data assignments

|  | $\boldsymbol{RMSE}$ | | | | $\boldsymbol{MAE}$ | | | | $\boldsymbol{R}^{\boldsymbol{2}}$ | | | |
| --- | --- | --- | --- | --- | --- | --- | --- | --- | --- | --- | --- | --- |
|  | *Training* | *STD* | *Testing* | *STD* | *Training* | *STD* | *Testing* | *STD* | *Training* | *STD* | *Testing* | *STD* |
| MLR | 1.149 | 0.005 | 1.151 | 0.019 | 0.845 | 0.003 | 0.846 | 0.012 | 0.629 | 0.003 | 0.628 | 0.010 |
| RR | 1.149 | 0.005 | 1.151 | 0.019 | 0.846 | 0.003 | 0.847 | 0.012 | 0.629 | 0.003 | 0.628 | 0.010 |
| LR | 1.887 | 0.007 | 1.887 | 0.027 | 1.436 | 0.005 | 1.435 | 0.020 | 0.000 | 0.000 | 0.000 | 0.001 |
| RFR | 0.497 | 0.004 | 0.797 | 0.021 | 0.322 | 0.002 | 0.518 | 0.009 | 0.931 | 0.001 | 0.822 | 0.010 |
| GBR | 0.961 | 0.005 | 0.988 | 0.018 | 0.702 | 0.004 | 0.718 | 0.011 | 0.741 | 0.003 | 0.726 | 0.009 |
| KNNR | 0.743 | 0.005 | 0.905 | 0.020 | 0.502 | 0.003 | 0.616 | 0.010 | 0.845 | 0.002 | 0.770 | 0.010 |


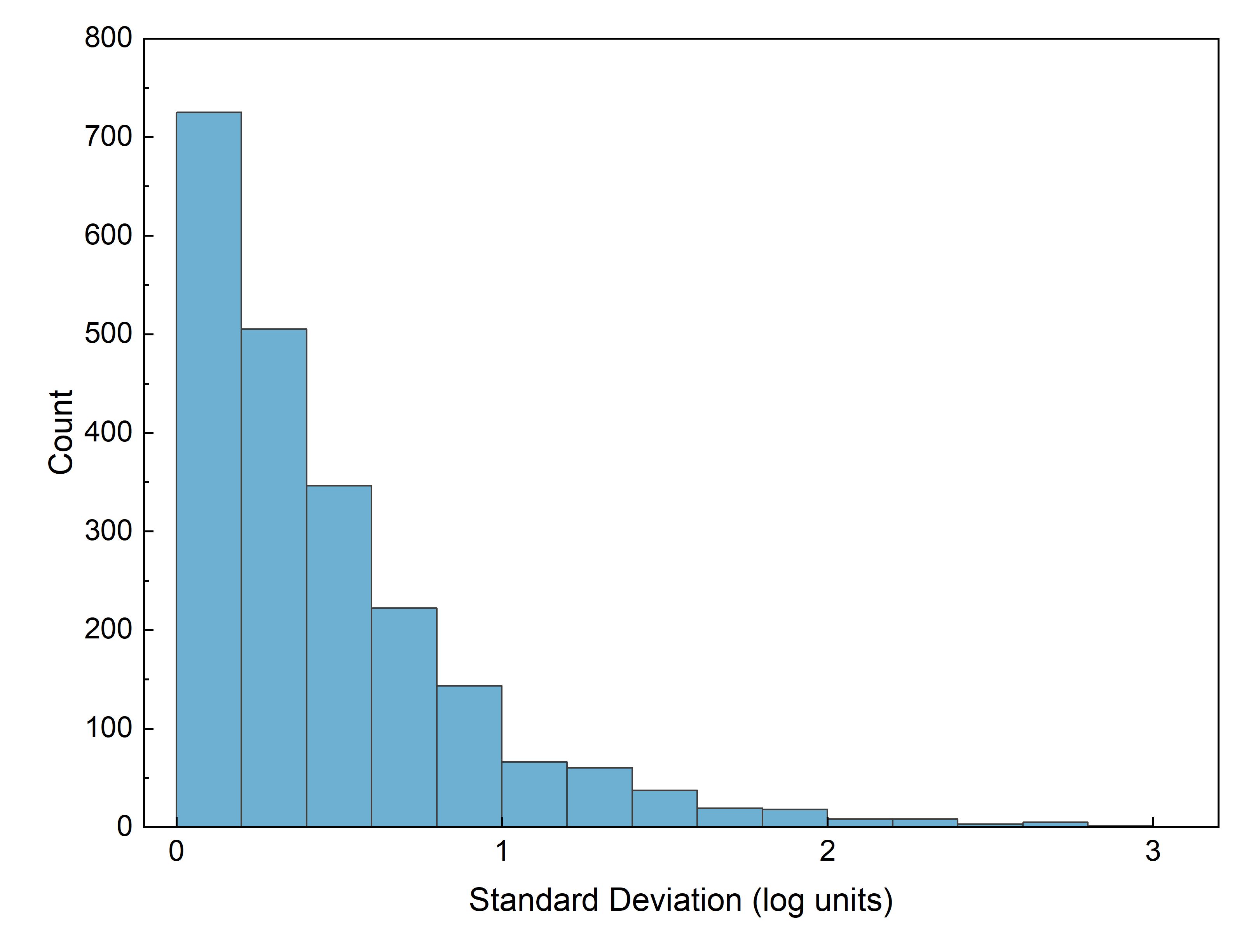


**Figure S18.** Histogram of isomer deviations for molecular formulas pertaining to two or more compounds within the dataset. The values range from 0 to 2.87 log units with an average value of 0.46 log units.

**
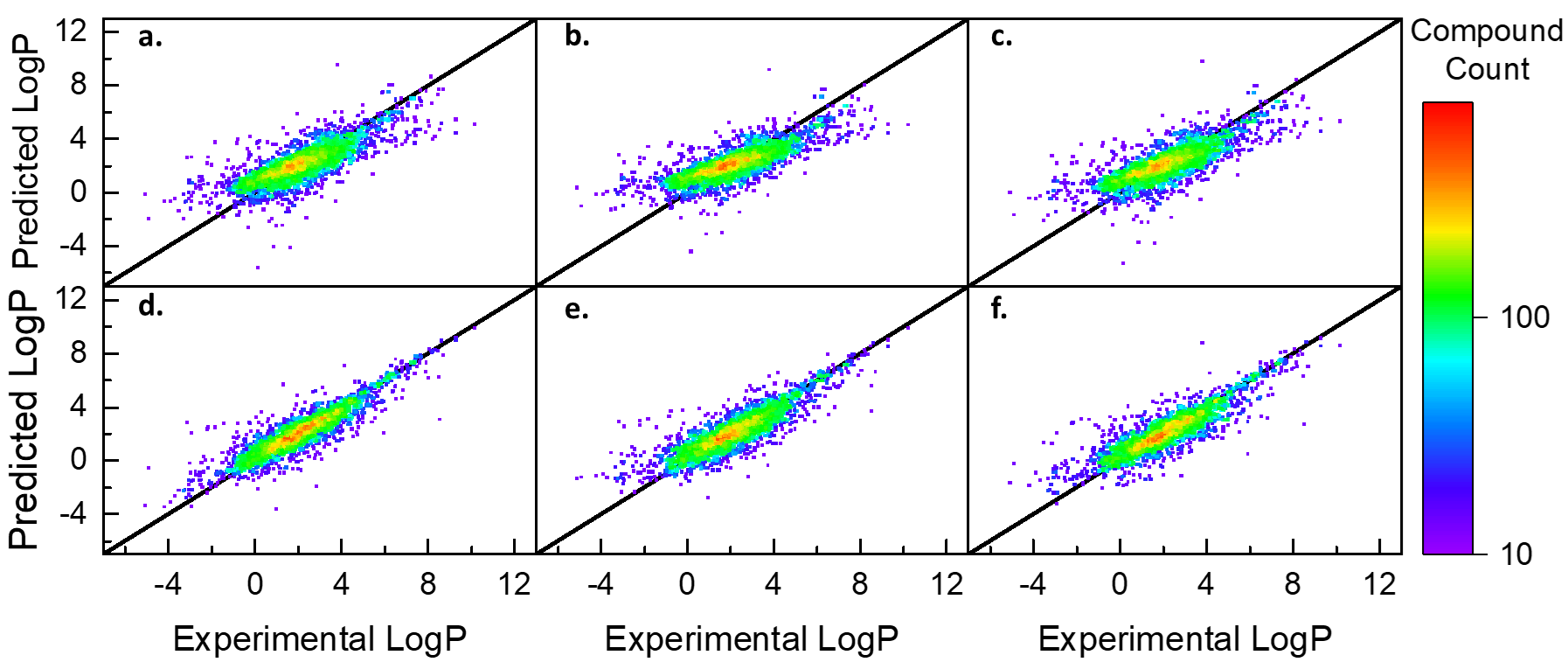
**

**Figure S19.** Parity density plots for experimentally determined and predicted values of the validation data (N = 3,084) for each of the six regression models with feature engineering and tuned hyperparameters. The linear models, except for Lasso, appear to have a similar performance while the RFR shows a better visual fit and higher density of points along y = x baseline. (**a**) Linear, (**b**) Ridge, (**c**) Lasso, (**d**) Random Forest, (**e**) Gradient Boosted, (**f**) k-Nearest Neighbors

**Table S5.** Averaged values for $RMSE$, $MAE$, and $R^{2}$ for each model with feature engineering and tuned hyperparameters over 100 iterations of different training and validation data assignments

|  | $\boldsymbol{RMSE}$ | | | | $\boldsymbol{MAE}$ | | | | $\boldsymbol{R}^{\boldsymbol{2}}$ | | | |
| --- | --- | --- | --- | --- | --- | --- | --- | --- | --- | --- | --- | --- |
|  | *Training* | *STD* | *Testing* | *STD* | *Training* | *STD* | *Testing* | *STD* | *Training* | *STD* | *Testing* | *STD* |
| MLR | 1.129 | 0.004 | 1.131 | 0.018 | 0.835 | 0.003 | 0.837 | 0.010 | 0.643 | 0.003 | 0.640 | 0.012 |
| RR | 1.157 | 0.004 | 1.157 | 0.019 | 0.856 | 0.003 | 0.857 | 0.011 | 0.625 | 0.003 | 0.623 | 0.011 |
| LR | 1.142 | 0.004 | 1.143 | 0.018 | 0.843 | 0.003 | 0.845 | 0.010 | 0.634 | 0.003 | 0.632 | 0.012 |
| RFR | 0.561 | 0.003 | 0.780 | 0.018 | 0.377 | 0.002 | 0.524 | 0.009 | 0.912 | 0.001 | 0.829 | 0.008 |
| GBR | 0.789 | 0.004 | 0.879 | 0.019 | 0.574 | 0.003 | 0.628 | 0.010 | 0.825 | 0.002 | 0.782 | 0.011 |
| KNNR | 0.758 | 0.005 | 0.876 | 0.018 | 0.520 | 0.003 | 0.604 | 0.009 | 0.839 | 0.002 | 0.784 | 0.009 |

# **S3.3. Comparing Predictions by Compound Class**


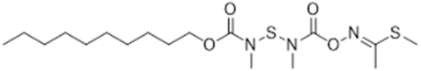

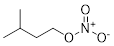

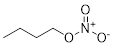

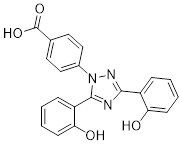

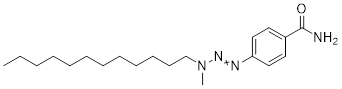

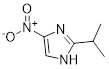

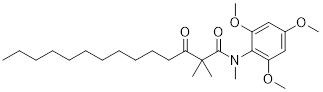

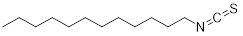

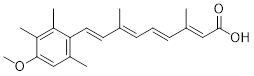

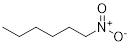

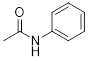

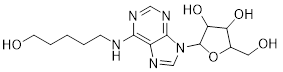

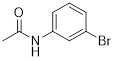

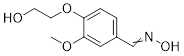

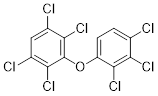

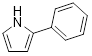

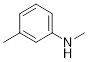

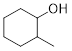

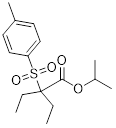

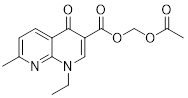


**(a)**

**(b)**

**(c)**

**(d)**

**(e)**

**(h)**

**(n)**

**(i)**

**(o)**

**(j)**

**(p)**

**(k)**

**(q)**

**(r)**

**(l)**

**(m)**

**(f)**

**(g)**

**(s)**

**(t)**

**Figure S20**. Twenty compounds identified as having the (**RED**) top ten largest absolute errors and (**GREEN)** top ten smallest absolute errors between the experimental partition coefficients and the MF-LOGP predicted values

**Table S6.** Experimental and predicted partition coefficients for the compounds identified to have the ten largest and smallest absolute error between experimental and predicted partition coefficients

| **Index** | **IUPAC name** | **Exp** $\boldsymbol{LogP}$ | **Pred** $\boldsymbol{LogP}$ | **Difference** |
| --- | --- | --- | --- | --- |
| **(a)** | methyl (1E)-N-[[decoxycarbonyl(methyl)amino]sulfanyl-methylcarbamoyl]oxyethanimidothioate | 6.20 | 0.19 | 6.01 |
| **(b)** | 3-Methylbutyl nitrate | 2.84 | -3.05 | 5.89 |
| **(c)** | Butyl nitrate | 2.15 | -2.87 | 5.02 |
| **(d)** | 4-[3,5-bis(2-hydroxyphenyl)-1,2,4-triazol-1-yl]benzoic acid | 6.30 | 1.97 | 4.33 |
| **(e)** | 4-(dodecyl-methylamino)diazenylbenzamide | 6.58 | 2.78 | 3.80 |
| **(f)** | 2-Isopropyl-4-nitro-1H-imidazole | 0.90 | -2.75 | 3.65 |
| **(g)** | N,2,2-trimethyl-3-oxo-N-(2,4,6-trimethoxyphenyl)tetradecanamide | 6.67 | 3.02 | 3.65 |
| **(h)** | 1-Isothiocyanatododecane | 7.13 | 3.77 | 3.36 |
| **(i)** | (2E,4E,6E,8E)-9-(4-methoxy-2,3,6-trimethylphenyl)-3,7-dimethylnona-2,4,6,8-tetraenoic acid | 6.40 | 3.13 | 3.27 |
| **(j)** | 1-Nitrohexane | 2.70 | -0.53 | 3.23 |
| **(k)** | N-phenylacetamide | 4.94 | 4.94 | 2.68E-4 |
| **(l)** | 2-(hydroxymethyl)-5-[6-(5-hydroxypentylamino)purin-9-yl]oxolane-3,4-diol | -0.24 | -0.24 | 3.33E-4 |
| **(m)** | N-(3-bromophenyl)acetamide | 2.31 | 2.31 | 3.58E-4 |
| **(n)** | 2-[4-(hydroxyiminomethyl)-2-methoxyphenoxy]ethanol | 0.82 | 0.82 | 5.17E-4 |
| **(o)** | [2,2',3,3',4,5',6'-Heptachlorodiphenyl ether](https://www.ncbi.nlm.nih.gov/pcsubstance/?term=%222%2C2%27%2C3%2C3%27%2C4%2C5%27%2C6%27-Heptachlorodiphenyl%20ether%22%5bCompleteSynonym%5d%20AND%2093479%5bStandardizedCID%5d) | 7.14 | 7.14 | 8.38E-4 |
| **(p)** | 2-Phenyl-1H-pyrrole | 2.76 | 2.76 | 2.37E-3 |
| **(q)** | N,3-Dimethylaniline | 2.19 | 2.19 | 2.36E-3 |
| **(r)** | 2-Methylcyclohexanol | 1.84 | 1.84 | 3.60E-3 |
| **(s)** | propan-2-yl 1-(4-methylphenyl)sulfonylcyclohexane-1-carboxylate | 3.21 | 3.21 | 3.91E-3 |
| **(t)** | acetyloxymethyl 1-ethyl-7-methyl-4-oxo-1,8-naphthyridine-3-carboxylate | 1.21 | 1.21 | 4.44E-3 |


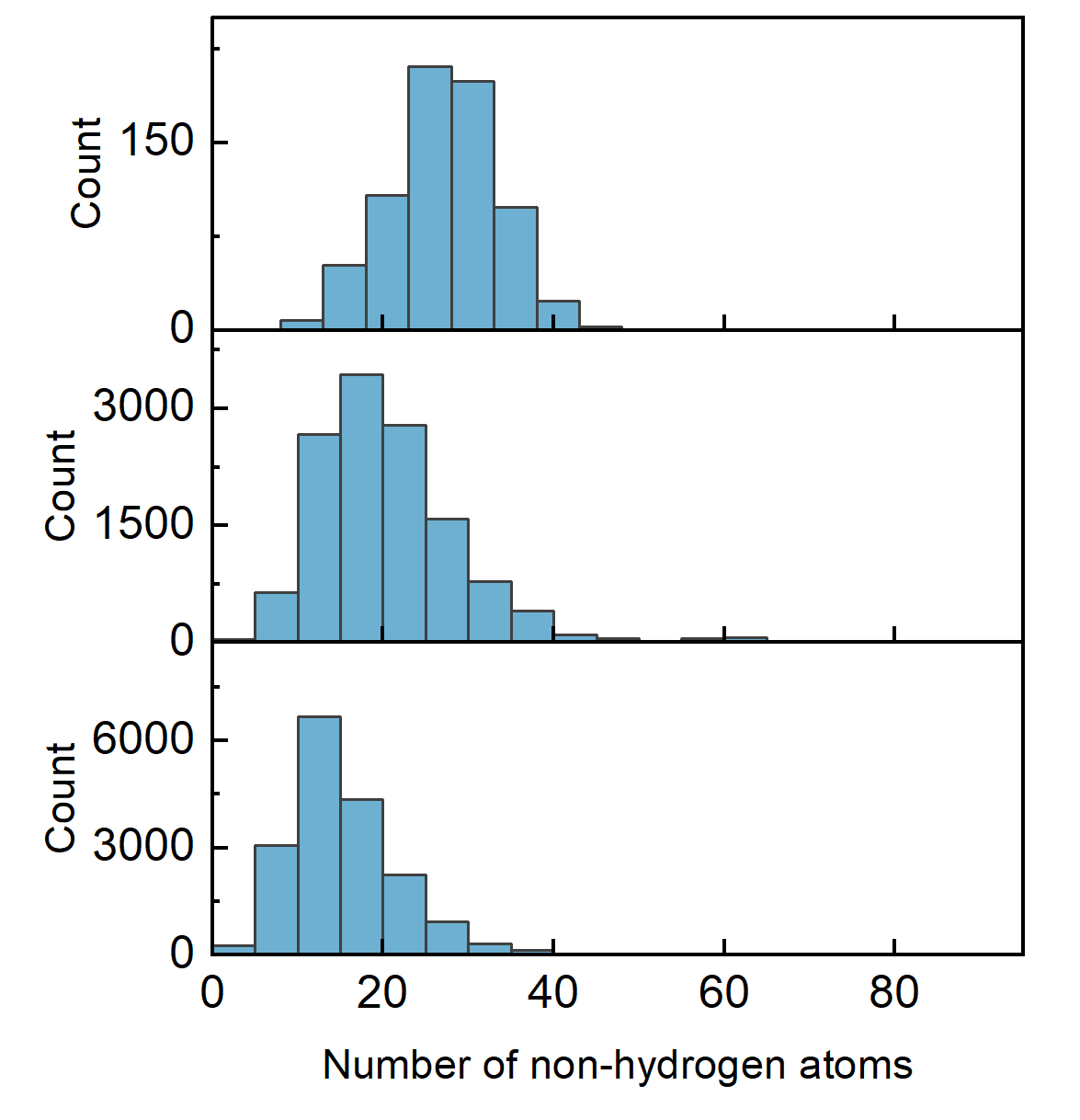


a.

b.

c.

**Figure S21.** Occurrences of compounds as a function of the total number of non-hydrogen atoms in (**a**) Plante et al., (**b**) Ulrich et al., and (**c**) MF-LOGP dataset

Table S7. Analysis of MF-LOGP on Ulrich et al. and Plante et al. datasets.

|  | Ulrich et al. | Plante et al. |
| --- | --- | --- |
| RMSE | 1.18 | 2.05 |
| MAE | 0.87 | 1.72 |
| R^2^ | 0.78 | 0.38 |
